# Improving Transformation of *Staphylococcus aureus* Belonging to the CC1, CC5 and CC8 Clonal Complexes

**DOI:** 10.1371/journal.pone.0119487

**Published:** 2015-03-25

**Authors:** Mary Janice Jones, Niles P. Donegan, Irina V. Mikheyeva, Ambrose L. Cheung

**Affiliations:** 1 Department of Microbiology and Immunology, Geisel School of Medicine at Dartmouth, Hanover, New Hampshire, United States of America; 2 Department of Biochemistry, Geisel School of Medicine at Dartmouth, Hanover, New Hampshire, United States of America; Rockefeller University, UNITED STATES

## Abstract

Methicillin resistant *Staphylococcus aureus* (MRSA) is an opportunistic pathogen found in hospital and community environments that can cause serious infections. A major barrier to genetic manipulations of clinical isolates has been the considerable difficulty in transforming these strains with foreign plasmids, such as those from *E*. *coli*, in part due to the type I and IV Restriction Modification (R-M) barriers. Here we combine a Plasmid Artificial Modification (PAM) system with DC10B *E*. *coli* cells (*dcm* mutants) to bypass the barriers of both type I and IV R-M of *S*. *aureus*, thus allowing *E*. *coli* plasmid DNA to be transformed directly into clinical MRSA strains MW2, N315 and LAC, representing three of the most common clonal complexes. Successful transformation of clinical *S*. *aureus* isolates with *E*. *coli*-derived plasmids should greatly increase the ability to genetically modify relevant *S*. *aureus* strains and advance our understanding of *S*. *aureus* pathogenesis.

## Introduction

*Staphylococcus aureus* is a common microorganism that colonizes asymptomatically 20–50% of the human population [1,2]. *S*. *aureus* can cause superficial infections such as cellulitis and skin abscesses as well as deep-seated infections such as pneumonia, endocarditis and sepsis [3]. As an opportunistic pathogen, MRSA is one of the leading causes of hospital acquired (HA-MRSA) infections [4]. Community acquired MRSA (CA-MRSA) cases, typically found in healthy human populations, have also been reported with increased frequency [5]. To understand *S*. *aureus* pathogenesis in the clinical context, it is important to be able to genetically manipulate the clinically-relevant organisms. However, this is hindered by the considerable difficulty in transforming clinical *S*. *aureus* isolates, especially with exogenous DNA from *E*. *coli*. The reason for this difficulty is due to several restriction barriers present in *S*. *aureus* that preclude easy transformation of DNA from *E*. *coli*. To overcome this barrier, *S*. *aureus* strain RN4220 has been used as an intermediary [6]. RN4220 has an impaired ability to restrict foreign DNA, thus allowing *E*. *coli*-derived plasmid DNA to be properly methylated and subsequently used to transform clinically relevant *S*. *aureus* isolates. Despite using this intermediary, there are still clinical strains that are difficult to transform [7], in part due to additional restriction systems that cannot be easily overcome by passage through the intermediary *S*. *aureus* strain RN4220.

The main barriers to transformation in many bacteria are the Restriction Modification (R-M) systems that recognize and restrict foreign DNA [8]. Currently, there are four known R-M system, three of which, types I, II and IV, are likely to be present in *S*. *aureus* [8–11]. In *S*. *aureus* and other microbes, the type I R-M system is encoded by three genes, *hsdR* (restriction), *hsdS* (specificity) and *hsdM* (modification) [12]. The products of these genes form complexes of either R_2_M_2_S or M_2_S [12]. The M_2_S complex is responsible for methylating adenines in DNA in a specific pattern that can be recognized by the *hsdR* gene product. The R_2_M_2_S complex is responsible for recognizing non-methylated DNA as foreign and restricting it [12]. In the nine sequenced *S*. *aureus* strains, there are two M_2_S complexes, broadly termed as *hsdMS* [9], and in this paper specified as *hsdMS*-1 and *hsdMS*-2. The target recognition sites (TRS) of the *hsdMS*-1 and *hsdMS*-2 systems are specific to individual clonal complexes [13]. The *hsdM* and *hsdR* genes appear to be conserved in *S*. *aureus* [9], while the *hsdS* genes share much less homology among various clonal complexes, which represent an appellation denoting common genotype and lineage. However, *hsdS* is conserved within each clonal complex, suggesting specificity of the R-M system within the same clonal complex [9]. The chemically mutagenized *S*. *aureus* strain RN4220 (which acts as an intermediary between *E*. *coli* and other *S*. *aureus* strains), which belongs to *S*. *aureus* CC8, has a mutation in their *hsdR* genes (called *sau1hsdR*) that renders the *hsdR* gene product non-functional [9]. This mutation in *hsdR* allows *E*. *coli* DNA to be transformed into *S*. *aureus* strains lacking a functional *hsdR*. Nonetheless, transformation of these *S*. *aureus* strains remains difficult [14] [15], thus implying that bypassing the type I R-M system alone is not enough for efficient transformation. *hsdMS*-1 and *hsdMS*-2 are located on the νSaα and νSaβ pathogenicity islands, respectively [16], which are formerly mobile elements on the *S*. *aureus* chromosome, each composed of several divergent subclasses. Both the *hsdMS* systems and their surrounding pathogenicity island cluster phenotypically with the clonal complex designation [16], suggesting that *hsd*-based TRS diversity was set down as these νSa genetic elements established themselves in various *S*. *aureus* populations.

The type II R-M system consists of DNA methylases and restriction endonuclease enzymes (REases), similar to those commonly used in molecular genetic laboratories [17]. There are at least 11 subclasses of type II R-M enzymes [18], all of which recognize specific DNA sequences and digest the DNA at either specific points or a pre-determined distance away from the recognition site to yield restricted fragments [19] [18]. There are two type II R-M enzymes in *S*. *aureus*, SauAI, which recognizes the double stranded GATC site [11] and Sau961, which recognizes the DNA sequence GGNCC [20]. However, these REases are only found in a small number of *S*. *aureus* strains [11],[20]. Interestingly, DNA passaged through *E*. *coli* can bypass the type II R-M barrier in *S*. *aureus* because *E*. *coli* Dam methylase methylates the adenine of the DNA sequence GATC [21,22] while Dcm methylase modifies the cytosine of CC(A/T)GG [10,22], essentially producing a methylation pattern on *E*. *coli* DNA similar to that of *S*. *aureus*.

While the type III R-M system cannot be easily discerned in *S*. *aureus*, they are found in other Gram positive bacteria [23]. This R-M system consists of the *mod* and *res* genes, with their gene products forming a complex that recognizes, modifies and restricts foreign DNA. The DNA must be in a head-to-head, inverse configuration to be recognized and restricted by the type III R-M system [18] [24].

Lastly, SauUSI, representing the type IV R-M system in *S*. *aureus*, is conserved in all sequenced genomes of *S*. *aureus*. It functions by recognizing and then cleaving methylated cytosines, specifically 5-methylcytosine and 5-hydroxymethylcytosine [10]. Practically, this is observed as a barrier to direct transformation of *S*. *aureus* with plasmids from *E*. *coli* since the *E*. *coli dcm* system produces a particular pattern of cytosine methylation recognized as foreign by SauUSI in *S*. *aureus* [10]. To bypass the SauUSI restriction, Δ*dcm E*. *coli* called DC10B which does not methylate these cytosines was created [25]. Plasmids obtained from DC10B can be used to directly transform *S*. *aureus*. Interestingly, *S*. *aureus* RN4220 and strains belonging to the CC5 complex (i.e. Mu50, Mu3 and N315) have mutations in *sauUSI* that result in loss of SauUSI function. These strains are therefore able to readily accept plasmid DNA from *E*. *coli* [14].

In order to bypass both R-M I and IV barriers, and to evaluate the roles that clonal-complex specific genes such as *hsdMS*-1 and *hsdMS*-2 play in restriction barriers in *S*. *aureus*, we engineered DC10B cells to encode a Plasmid Artificial Modification (PAM) system [26] of either the HsdMS-1 or HsdMS-2 complexes specific to three dominant *S*. *aureus* clonal complexes, CC1, CC5 and CC8 [27]. We then evaluated the proficiency of these PAM systems to enable shuttle plasmids pEPSA5 and pSK236 from *E*. *coli* to be directly transformed into *S*. *aureus*, instead of via an intermediary *S*. *aureus* strain.

## Materials and Methods

### Bacterial strains and culture conditions

Table 1 contains a list of bacterial strains used in these studies. *S*. *aureus* strains were routinely grown in Tryptic Soy Broth (TSB) and the *E*. *coli* strains were grown in Luria-Bertani (LB) broth. All strains were grown at 37°C while shaking at 250 rpm. The cell densities were measured in borosilicate glass tubes with an 18 mm diameter, using absorbance (OD) at 650nm (unless otherwise noted) in a Spectronic 20D+ spectrophotometer. Ampicillin (Amp) (50 μg/ml), chloramphenicol (Cm) (10 μg/ml for *S*. *aureus* strains, 30 μg/ml for *E*. *coli* strains), or kanamycin (Kan) (40 μg/ml) were added to the appropriate media as required by a particular strain. To amplify low copy number plasmids, overnight cultures of strains with pEPSA5 were diluted by half with warm LB and treated with 125 μg/mL of chloramphenicol and grown for several hours at 37°C with shaking at 250 rpm [28].

**Table 1:** Bacterial Strains and Plasmids Used in Study.

Bacterial Strains or Plasmids	Description or Genotype	Source or reference
*Plasmids*		
pCR2.1-AmpS	Cloning plasmid. ColE1 origin of replication in *E*. *coli*; Kan^R^, Amp^S^	This study
pEPSA5	Shuttle plasmid. p15A origin of replication in *E*. *coli*; Amp^R^ in *E*. *coli*, Cm^R^ in *S*. *aureus*. 6852bp.	[29]
pACYC184	Cloning plasmid. p15A origin of replication in *E*. *coli*; Tet^R^ and Cm^R^	[30]
pSK236	Shuttle plasmid. ColE1 origin of replication in *E*. *coli*; Amp^R^ in *E*. *coli*, Cm^R^ in *S*. *aureus*. 5597 bp.	[31]
*Strains*		
*S*. *aureus* N315	USA100, MRSA, CC5	[32]
*S*. *aureus* MW2	USA400, MRSA, CC1	[33]
*S*. *aureus* USA300 LAC	USA300 LAC, MRSA, CC8	[34]
*E*. *coli* XL-1 Blue	*E*. *coli* K12; Tet^R^	Agilent Technologies
*E*. *coli* DC10B	*E*. *coli* K12, Δ*dcm* mutant	[25]
*E*.*coli* DC10B pCR2.1-Amp^S^::*hsdMS*-2^CC1^ (ALC7764)	*E*. *coli* K12, Δ*dcm* mutant with pCR2.1-Amp^S^ containing *hsdMS*-2^CC1^; Kan^R^	This study
*E*.*coli* DC10B pCR2.1-AmpS::*hsdMS*-2^CC5^ (ALC7762)	K12 *E*. *coli*, Δ*dcm* mutant with pCR2.1-Amp^S^ containing *hsdMS*-2^CC5^; Kan^R^	This study
*E*.*coli* DC10B pCR2.1-AmpS::*hsdMS*-2^CC8^ (ALC7884)	*E*. *coli* K12, Δ*dcm* mutant with pCR2.1-Amp^S^ containing *hsdMS*-2^CC8^; Kan^R^	This study
*E*.*coli* DC10B pCR2.1-AmpS::*hsdMS*-1^CC1^ (ALC7765)	*E*. *coli* K12, Δ*dcm* mutant with pCR2.1-Amp^S^containing *hsdMS*-1^CC1^; Kan^R^	This study
*E*.*coli* DC10B pCR2.1-AmpS::*hsdMS*-1^CC5^ (ALC7763)	*E*. *coli* K12, Δ*dcm* mutant with pCR2.1-Amp^S^ containing *hsdMS*-1^CC5^; Kan^R^	This study
*E*.*coli* DC10B pCR2.1-AmpS::*hsdMS*-1^CC8^ (ALC7930)	*E*. *coli* K12, Δ*dcm* mutant with pCR2.1-Amp^S^ containing *hsdMS*-1^CC8^; Kan^R^	This study
*E*.*coli* DC10B pACYC184::*hsdMS*-2^CC1^ (ALC7760)	*E*. *coli* K12, Δ*dcm* mutant with pACYC184 containing *hsdMS*-2^CC1^; Cm^R^	This study
*E*.*coli* DC10B pACYC184::*hsdMS*-2^CC5^ (ALC7758)	*E*. *coli* K12, Δ*dcm* mutant with pACYC184 containing *hsdMS*-2^CC5^; Cm^R^	This study
*E*.*coli* DC10B pACYC184::*hsdMS*-2^CC8^ (ALC7885)	*E*. *coli* K12, Δ*dcm* mutant withpACYC184 containing *hsdMS*-2^CC8^; Cm^R^	This study
*E*.*coli* DC10B pACYC184::*hsdMS*-1^CC1^ (ALC7761)	*E*. *coli* K12, Δ*dcm* mutant with pACYC184 containing *hsdMS*-1^CC1^; Cm^R^	This study
*E*.*coli* DC10B pACYC184::*hsdMS*-1^CC5^ (ALC7759)	*E*. *coli* K12, Δ*dcm* mutant with pACYC184 containing *hsdMS*-1^CC5^; Cm^R^	This study
*E*.*coli* DC10B pACYC184::*hsdMS*-1^CC8^ (ALC7929)	*E*. *coli* K12, Δ*dcm* mutant with pACYC184 containing *hsdMS*-1^CC8^; Cm^R^	This study

### DNA manipulations

*E*. *coli* plasmid purification was performed using Omega E.Z.N.A. miniprep kits per the manufacturer’s instructions, while plasmid isolation from *S*. *aureus* was performed as described previously [35]. Plasmid transformations in *S*. *aureus* were achieved using electro-competent *S*. *aureus* cells made similarly to Löfblom *et al* [36] and Monk *et al* [25]. Briefly, overnight cultures of *S*. *aureus* N315, MW2 or USA300 LAC were diluted to an OD_578_ of 0.500 in pre-warmed TSB media. The cultures were grown at 37°C with shaking for 30 min, transferred to centrifuge tubes and chilled on ice for 10 min. The cells were harvested by centrifuging at 4000 x *g* at 4°C for 10 min, washed twice in equal volume ice-cold autoclaved water and pelleted at 4°C. The cells were then washed in 1/10 volume ice-cold 10% sterile glycerol, repeated with 1/25 volume ice-cold 10% sterile glycerol, resuspended in 1/200 volume of ice-cold 10% sterile glycerol and then aliquoted (50 μl) into tubes. Competent cells were made fresh the day of electroporation and stored on ice until needed. For electroporation, the cells were left at room temperature for 5 min, centrifuged at 5000 x g for 1 min and resuspended in 50 μl sterile 10% glycerol with 500 mM sucrose. 5 μl of Pellet Paint (Novagen) precipitated DNA was added to the cells in a sterile 0.2 cm electroporation cuvette. The cells were pulsed once using BioRad MicroPulser at 1.8kV and 2.5 msec time constant, outgrown in 1 ml of TSB/500 mM sucrose for 1 hour at 37°C, spread on TSA/Cm plates and incubated overnight at 37°C.

### Creating Plasmid Artificial Modification (PAM) Systems in DC10B Cells

The *hsdMS*-1 and *hsdMS*-2 operons (annotated in the N315 genome as *sa0391-2* and *sa1626-5*, respectively) were amplified from the chromosomal DNA of *S*. *aureus* strains MW2 (CC1), N315 (CC5) and USA300 LAC (CC8), using primers listed in Table 2 (MJJ-1—MJJ-9). As noted earlier [13], *hsdMS*-1 of CC5 and CC8 are homologous while *hsdMS*-2 of CC1 is homologous to that of CC8. However, the presence of several non-conserved mutations between these two homologous groups warrants investigation to determine if functional homology exists. Given that the promoters of both *hsdMS* systems have not been defined, and that these systems will be transcribed in *E*. *coli*, we chose a well-characterized *E*. *coli* promoter to drive *hsdMS* transcription. More specifically, the moderately constitutive sigma-70 *E*. *coli* P(bla) promoter (BBa_I14018), together with a consensus ribosome binding site (BBa_J61101), was chosen from the Registry of Standard Biological Parts [37] and cloned upstream of the *hsdM1* and *hsdM2* genes by PCR using the MJJ-1 or MJJ-2 primers respectively (Table 2).

**Table 2:** Oligonucleotides Used in Study.

Oligonucleotide	Sequence
MJJ-1	GTCGACTGTAAGTTTATACATAGGCGAGTACTCTGTTATGGAAAGACAGGACCCACTAGATGTCTATTACTGAAAAACAA
MJJ-2	GTCGACTGTAAGTTTATACATAGGCGAGTACTCTGTTATGGAAAGACAGGACCCACTAGATGATTTTGAAAGCATTTGAA
MJJ-3	GGCCGGATCCTTAAATAAACATTTTTTGTAA
MJJ-4	GCGCGGATCCTTAAACAAACATTTTTTGTAA
MJJ-5	GGCCGGATCCTCAAATAAACATTTTCTGTAA
MJJ-6	GGCCGGATCCTTATAAGAACATTTTTTGTA
MJJ-7	GGCCAATAATTACGAATAATAAAAA
MJJ-8	GGCCCTCGAGCTTAGACATTCACCCAATCCT
MJJ-9	GGCCCTCGAGAATAATTACGAATAATAAAAA
MJJ-10	GGCCCTTAGACATTCACCCAATCCT
pEPSA5F-qPCR	TCAAGACTAACTCCTCTAAATC
pEPSA5R-PCR	TGTCATTCCGCTGTTATG
pCR2.1F-qPCR	CCCGTCAAGCTCTAAATC
pCR2.1R-qPCR	GATAGGGTTGAGTGTTGTT
pACYC184F-qPCR	GAGAAGCAGGCCATTATC
pACYC184R-qPCR	CGAAGTTAGGCTGGTAAG
pSK236F-qPCR	TTCAGGAATTGTCAGATAGG
pSK236R-qPCR	GAGGCTCAACGTCAATAA

To facilitate antibiotic selection of other plasmids by ampicillin, the ampicillin resistance gene of pCR2.1 TOPO (Invitrogen) was disrupted by using *Bpm*I and *Bsa*I (New England Biolabs) followed by blunt-end ligation. Ampicillin sensitivity of the resulting plasmid, named pCR2.1-AmpS, was verified by plating the strain on LB/kanamycin and LB/ampicillin agar.

After PCR amplification, the amplified *hsdMS*-1 and *hsdMS*-2 DNA were then digested with *Sal*I and *Bam*HI, ligated to similarly digested pACYC184 or pCR2.1-AmpS using T4 ligase (New England Biolabs), and transformed into competent *E*. *coli* XL-1 Blue cells. Correct transformants were verified by restriction digestion and sequencing of the insertion site. Recombinant plasmids containing *hsdMS*-1 or *hsdMS*-2 were then used to transform *E*. *coli* DC10B cells and plated onto LB Agar with ampicillin (for pACYC184) or with kanamycin (for pCR2.1-AmpS). These resulting strains and plasmids are listed in Table 1, and are available upon request from our laboratory.

Competent aliquots of each of 12 resulting DC10B strains containing pCR2.1-AmpS/*hsdMS* or pACYC184/*hsdMS* were prepared and transformed with shuttle plasmid pEPSA5 or pSK236, respectively. The transformed DC10B cells were plated on LB supplemented with ampicillin and kanamycin (DC10B with pCR2.1-AmpS/*hsdMS* + pEPSA5) or ampicillin and chloramphenicol (DC10B with pACYC184/*hsdMS* + pSK236).

### Determining ratio of plasmid::*hsdMS* to the shuttle plasmid and transformaton efficiency of the *hsdMS*-2 modified plasmids from *E*. *coli* into *S*. *aureus*

DNA quantification and purity was performed by measuring absorbance at 260nm and 280nm on a BioPhotometer (Eppendorf Inc.) to yield the total concentrations of both plasmids miniprepped from the DC10B cells. Real time quantitative PCR (qPCR) was done to determine the ratio of pCR2.1-AmpS/*hsdMS*-2 to pEPSA5 and the ratio of pACYC184/*hsdMS-2* to pSK236. The qPCR was done on a Roche 480 II Light Cycler using LightCycler 480 DNA SYBR Green I Master Mix following the manufacter’s specifications, cycling conditions, and the primers (annotated as qPCR) in Table 2. Samples and standard curves were run in duplicate. Standard curves using known copy numbers of pCR2.1-AmpS/*hsdMS*-2, pEPSA5, pACYC184/*hsdMS*-2 and pSK236 were set up and the unknowns were quantified to the standard curve using the Light Cylcer 480SW software, version 1.5.1.62. The correct amplification products were verified via melt curve analysis. Using this information, the ratios of plasmids were determined and the concentrations of pEPSA5 and pSK236 in the plasmid samples could be established.

For transformations of *E*. *coli* plasmids into *S*. *aureus*, varying concentrations or amounts of pEPSA5 or pSK236, methylated by the *hsdMS*-2 system in DC10B cells, were transformed into respective *S*. *aureus* strains of the same clonal complex to create a curve to identify the amount of plasmid needed to successfully transform the recipient *S*. *aureus* bacteria. Pellet paint-precipitated DNA was used to transform *S*. *aureus* cells as described above.

### Verification of correct plasmids

To verify that the transformants contained pEPSA5 or pSK236, 10 colonies from each transformation were prepared for plasmid-prep, digested with *Eco*RI to linearize the DNA and then resolved on a 0.8% agar gel to confirm the correct size of the plasmid. DNA sequence analysis was also performed on selected plasmids to verify plasmid identity. All samples tested contained the correct plasmid.

### Statistical analysis

All statistical analysis was performed using GraphPad Prism, version 6.0c.

## Results

### Strategy for construction of PAM system strains

**Fig 1:**
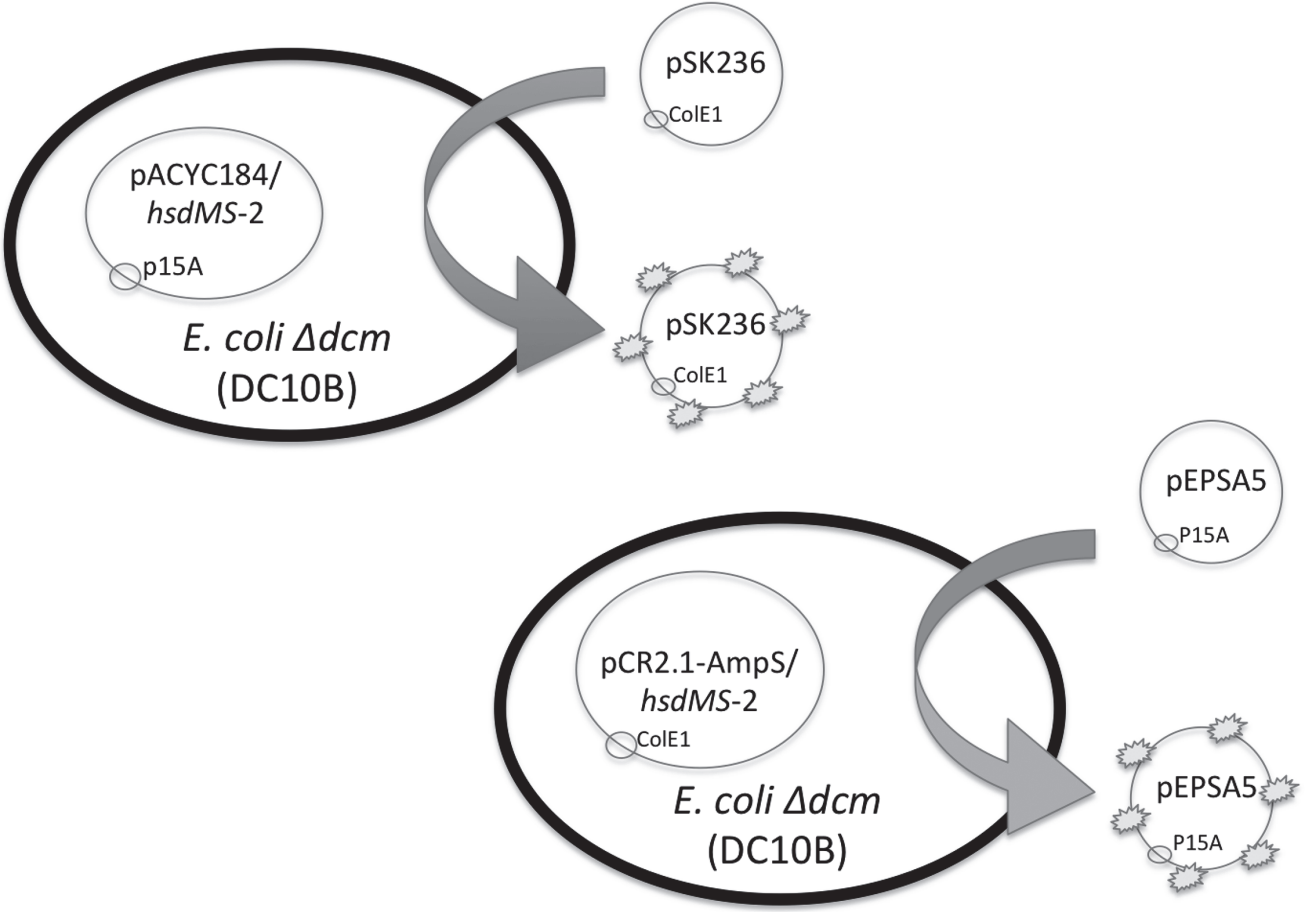
A pictorial of the genetic systems that protect DNA from the type I and type IV restriction modification systems. Shuttle plasmids pSK236 and pEPSA5 are cloned into DC10B *E*. *coli* cells containing the HsdMS-1 or HsdMS-2 system-modification complex. Different HsdMS-1 or HsdMS-2 modification complexes were used, depending on the origin of replication of the shuttle plasmids. Once methylated by the HsdMS complex (represented by the star-like explosion on the plasmid in the figure), the plasmids were transformed into *S*. *aureus*.

To facilitate direct transformation of plasmid DNA from *E*. *coli* into *S*. *aureus*, we have constructed two different *E*. *coli* plasmid systems that bypass the type I and type IV restriction systems in clinical *S*. *aureus* isolates, thus enabling direct transformation of *S*. *aureus* by plasmids containing either the *E*. *coli* p15A (i.e. pEPSA5) or pUC (i.e. pSK236 and pMAD) backbones. The shuttle plasmid pEPSA5, chosen for its xylose inducible promoter that allows genes to be expressed *in trans* [29], has a p15A origin of replication and is compatible with the ColE1 origin of replication in pCR2.1-AmpS in *E*. *coli* DC10B cells. Likewise, pSK236, commonly used to introduce genes into homologous or heterologous *S*. *aureus* strains [31], has a ColE1 origin which is compatible with the p15A origin of replication present in pACYC184 in *E*. *coli*. For proper plasmid methylation, DC10B cells containing recombinant pCR2.1-AmpS with *hsdMS*-1 or *hsdMS*-2 were used to modify the pEPSA5 plasmid (Fig. 1). Similarly, DC10B cells containing recombinant pACYC184 with *hsdMS*-1 or *hsdMS*-2 were used to modify the pSK236 plasmid (Fig. 1). As the TRS (target recognition sequence) of the *hsdMS*-1 and *hsdMS*-2 modification systems are known, we have listed in Table 3 the number of TRS assigned to each clonal complex for both shuttle plasmids.

**Table 3:** Frequency of target recognition sites (TRS) in pSK236 and pEPSA5 based on HsdMS in different clonal complexes.

*S*. *aureus* strain and clonal complex	TRS Sequence (5’-3’) [13]	Number of TRS sites present in pSK236	Number of TRS sites present in pEPSA5
MW2, CC1	hsdMS-1 CC1	1	1
	CCAY(N)_5_TTAA		
	hsdMS-2 CC1	2	1
	CCAY(N)_6_TGT		
N315, CC5	hsdMS-1 CC5	1	2
	ATC(N)_5_CCT		
	hsdMS-2 CC5	0	1
	CCAY(N)_6_GTA		
USA300 LAC, CC8	hsdMS-1 CC5	1	2
	ATC(N)_5_CCT		
	hsdMS-2 CC1	2	1
	CCAY(N)_6_TGT		

The number of TRS sites present in each shuttle plasmid is listed. CC8 has high sequence homology with the TRS sequences listed [13].

(Y = C or T, N = any base).

### Transformation Efficiencies of *hsdMS*-1 vs. *hsdMS*-2

**Fig 2:**
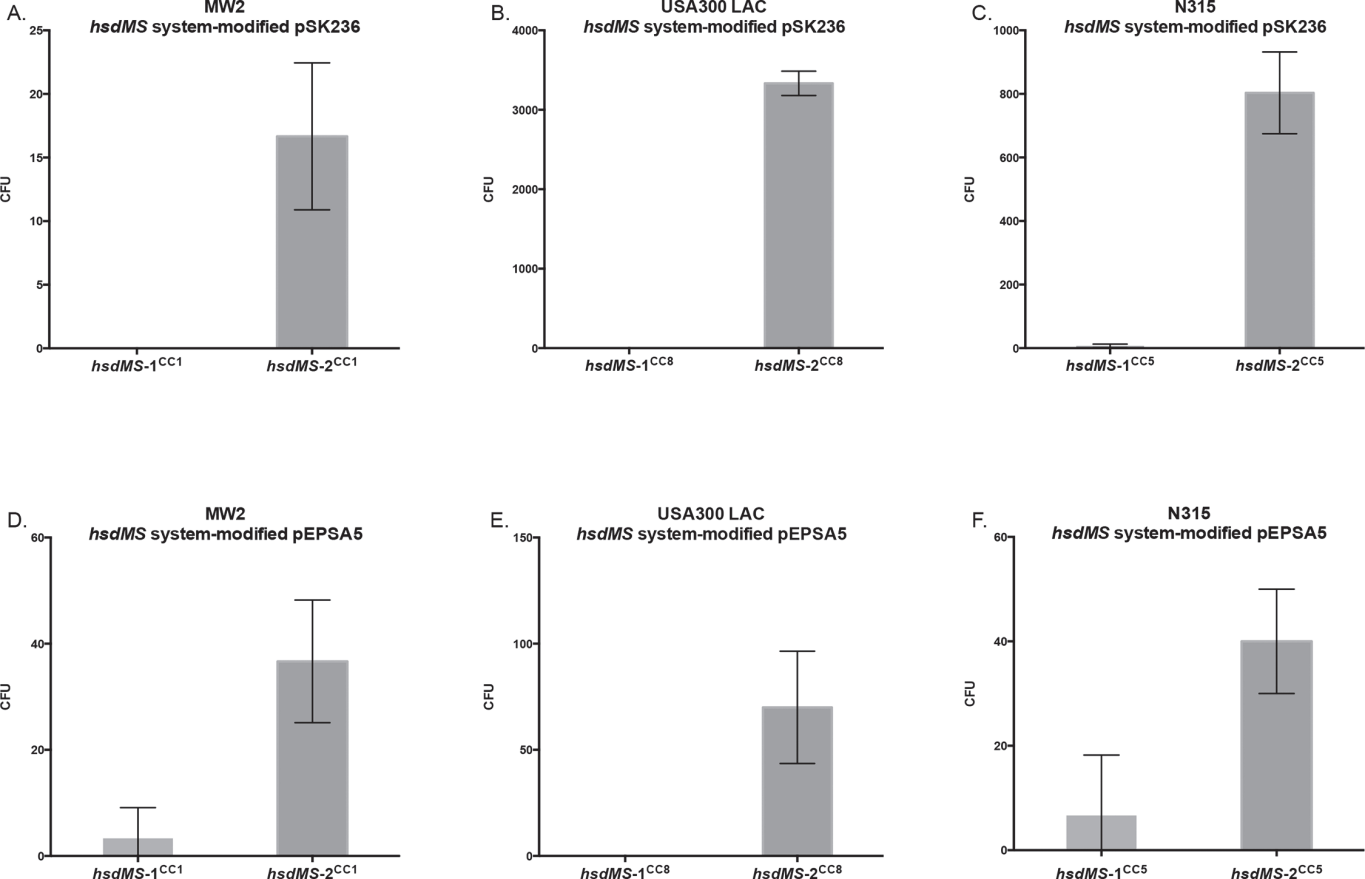
Differences in transformation efficiencies of *S*. *aureus* with pSK236 and pEPSA5 from *hsdMS*-1 and *hsdMS*-2 modification. A-C are for pSK236 while D-F are for pEPSA5. A and D pertain to MW2 (CC1), B and E are for USA300 LAC (CC8) and C and F correspond to N315 (CC5). For each, *S*. *aureus* was transformed with equal concentrations of plasmid DNA. The charts represent the mean number of transformants for each transformation (*n = 3)*, with the error bars representing the standard deviations. Paired t-tests were done for all experiments. With the exception of *hsdMS*-1 vs. *hsdMS*-2-modified pEPSA5 into N315, there was significant difference between HsdMS-1 and HsdMS-2 complexes (*p<0*.*05)* for all the remaining strains and plasmids.

*S*. *aureus* has two *hsdMS* operons. To determine which HsdMS complex is largely responsible for the proper methylation of plasmid DNA, we transformed *S*. *aureus* MW2 (CC1), N315 (CC5) and USA300 LAC (CC8), with pSK236 isolated from *E*. *coli* DC10B cells that contained either the recombinant plasmid pACYC184::*hsdMS*-1 or pACYC184::*hsdMS-2* corresponding to the matching clonal complex (e.g. *hsdMS*-1 and -2 from CC-1 into MW2, etc.). These three *S*. *aureus* strains were also transformed with recombinant pEPSA5 plasmids that had been modified in *E*. *coli* containing pCR2.1-AmpS::*hsdMS*-1 or pCR2.1-AmpS::*hsdMS*-2 corresponding to the appropriate clonal complex. The amounts of the plasmids transformed into *S*. *aureus* include both the shuttle plasmid and the *hsdMS*-2-modified plasmid. We also determined the ratio of *hsdMS2* plasmid to shuttle plasmid using quantitative qPCR with a standard curve. Notably, only the shuttle plasmids are capable of transforming *S*. *aureus* cells. The results showed that when normalized to the amount of shuttle plasmid present in the plasmid mix, *S*. *aureus* strains transformed with pEPSA5 or pSK236 methylated by the *hsdMS*-2 gene products resulted in more colonies than the corresponding strains transformed with pEPSA5 or pSK236 methylated by the *hsdMS*-1 gene products (Fig. 2). Specifically, *hsdMS*-2-modified pSK236 resulted in 73 and 516 times more colonies in strains N315 (Fig. 2C) and USA300 LAC (Fig. 2B), respectively, than *hsdMS*-1-modified pSK236. In MW2 (Fig. 2A), *hsdMS*-2-modified pSK236 resulted in ∼20 colonies whereas *hsdMS*-1-modified pSK236 resulted in no colonies. Using pEPSA5 as the shuttle vector, the transformation efficiency is generally less than that of pSK236, with the exception of strain MW2. In N315 (Fig. 2F) and MW2 (Fig. 2D), *hsdMS*-2-modified pEPSA5 resulted in 5 and 6 times more colonies, respectively, than *hsdMS*-1-modified pEPSA5. In USA300 LAC (Fig. 2E), *hsdMS*-2-modified pEPSA5 yielded over 50 transformants whereas *hsdMS*-1-modified pEPSA5 did not yield any transformants. Collectively, these data indicate that the HsdMS-2 complex, but not the HsdMS-1 complex, is responsible for the majority of type I R-M function in *S*. *aureus*.

### Measuring the effect of PAM on transformation of pSK236 and pEPSA5 into *S*. *aureus* strains MW2, USA300 LAC and N315

**Fig 3:**
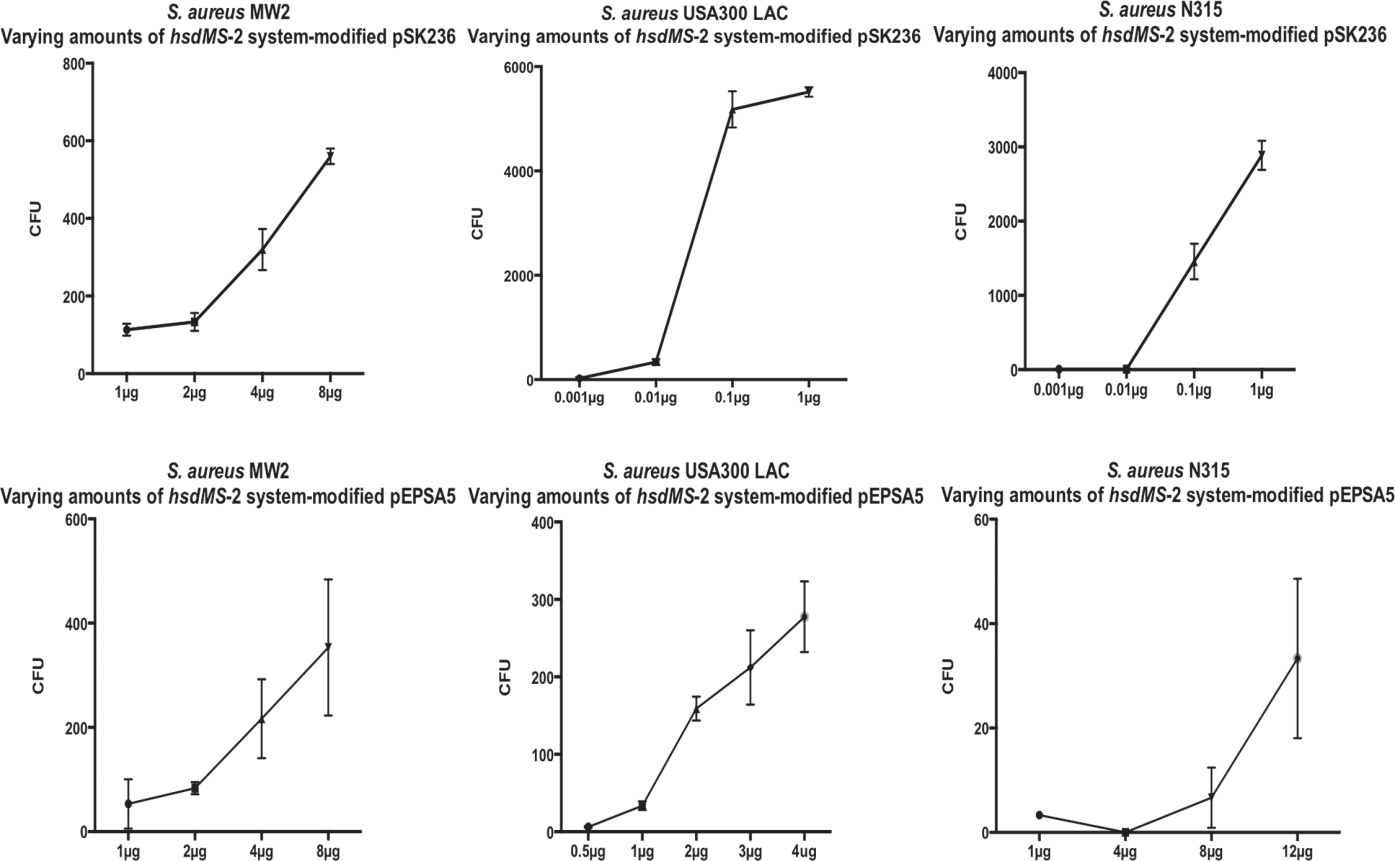
Graphs measuring transformation of *S*. *aureus* strains MW2, USA300 LAC and N315 with *hsdMS*-2 system-modification plasmids pSK236 and pEPSA5 (modified by *hsdMS*-2 in *E*. *coli* DC10B). Graphs A-C are for pSK236 efficiency, D-F are for pEPSA5 efficiency. A and D pertain to MW2 (CC1), B and E are for USA300 LAC (CC8) and C and F correspond to N315 (CC5). For each plasmid (*n = 3*), the error bars represent the standard deviation.

Recognizing that *hsdMS*-2 is responsible for the majority of type I R-M function in *S*. *aureus*, we next measured the effect of plasmid dosage on transformation efficiency using plasmids directly from *E*. *coli* and the amounts of shuttle plasmid DNA in the plasmid mix quantified by qPCR (see Table 4). In the case of *hsdMS*-2-modified pSK236, we showed that the number of transformants increased with plasmid dosage (Fig. 3A-C). Interestingly, while the transformation efficiency of *hsdMS*-2-modified pSK236 was high for USA300 LAC (Fig. 3B) and N315 (Fig. 3C) (5180 and 2880 colonies per μg of DNA), the transformation efficiency was not as high with pSK236 in MW2 (Fig. 3A), suggesting that MW2 may harbor additional restriction barrier(s). The transformation efficiency of *hsdMS*-2-modified pEPSA5 was generally less than that of *hsdMS*-2-modified pSK236. More specifically, the number of transformants plated from the transforming mixtures of *hsdMS*-2-modified pEPSA5 yielded ∼40 colonies per μg DNA for USA300 LAC (Fig. 3E) and ∼44 colonies per μg DNA for MW2 (Fig. 3D). The transformation efficiency of *hsdMS*-2-modified pEPSA5 for strain N315 (Fig. 3E) was low, yielding ∼3 colonies per μg of plasmid DNA from *E*. *coli*.

**Table 4:** The average ratio of pCR2.1-AmpS::*hsdMS*-2 to pEPSA5 and pACYC184::*hsdMS-2* to pSK236, as determined by qPCR.

	N315	MW2	USA300 LAC
pCR2.1-AmpS::*hsdMS*-2/ pEPSA5	19.6: 1	5: 1	3: 1
pACYC184::*hsdMS-2* /pSK236	4.1: 1	1: 1.6	1: 1.6

### Assessing the Effects of Bypassing Restriction Barriers I and IV

**Fig 4:**
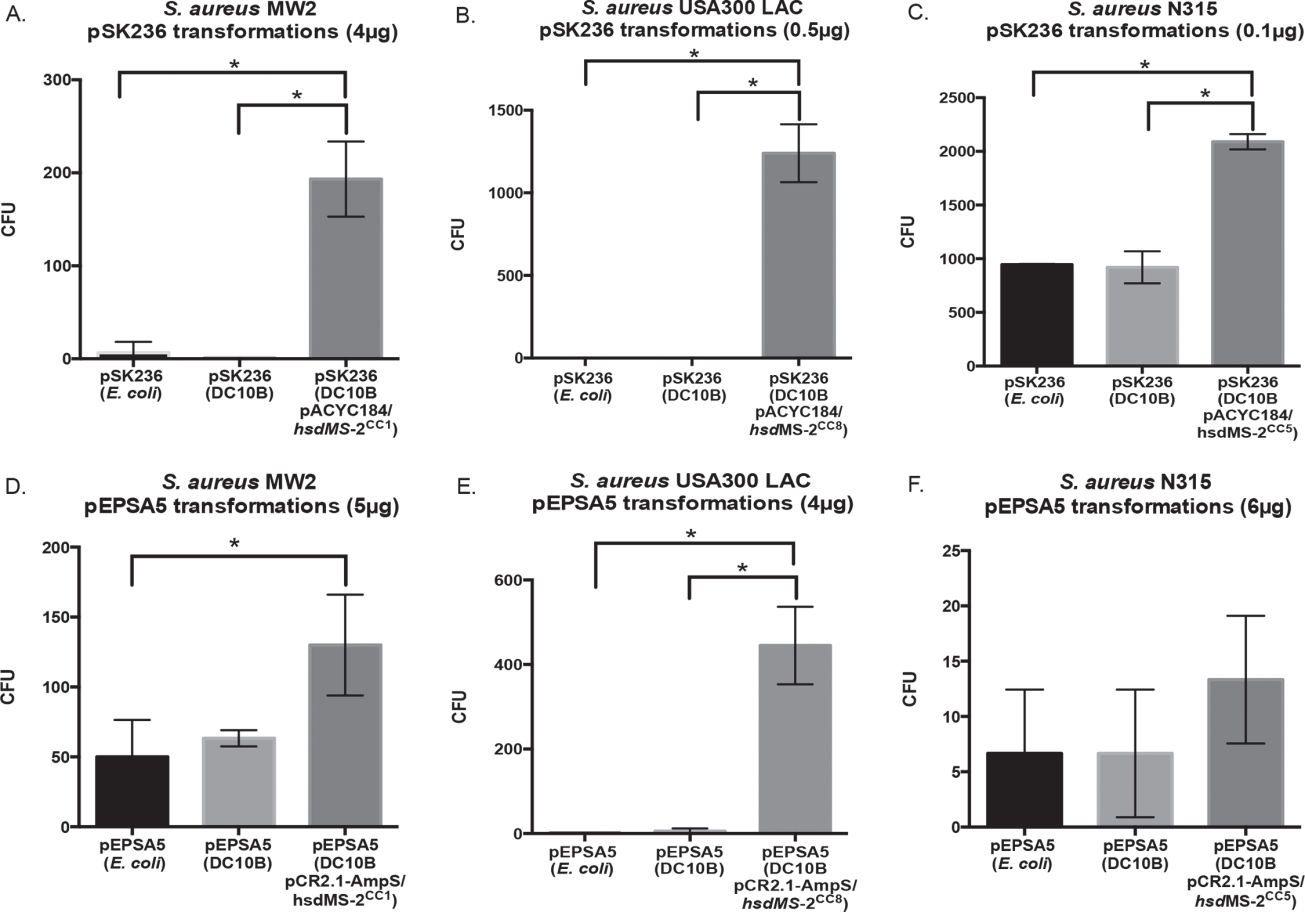
Transformation efficiencies of *S*. *aureus* strains using either pSK236 or pEPSA5 from *E*. *coli* K12, DC10B or from the DC10B *hsdMS*-2 system-modification complex. A-C represent data for pSK236 while D-F are for pEPSA5. A and D pertain to MW2 (CC1), B and E are for USA300 LAC (CC8) and C and F correspond to N315 (CC5). For each transformation, *n = 3*, except B (*n = 2*). The charts represent the mean of the number of transformants, with the error bars corresponding to standard deviations. * = Significant difference between transformations (*p*<0.05) as determined by Student’s t test.

To compare how our DC10B *hsdMS*-2-modified pEPSA5 and pSK236 improve transformation of *S*. *aureus* compared to pEPSA5 and pSK236 isolated from *E*. *coli* K12 or DC10B, equal amounts of pEPSA5 and pSK236 from all three *E*. *coli* sources were purified for each CC group and transformed into respective *S*. *aureus* strains (MW2, N315 or USA300 LAC). No major differences were observed by bypassing the type IV R-M restriction barrier when we compared plasmids from *E*. *coli* DC10B vs. those from K12 (Fig. 4). However, *hsdMS*-2-modified pSK236 yielded more transformed colonies than pSK236 obtained from *E*. *coli* DC10B or *E*. *coli* K12 in all three strains. Analysis via paired t-test showed that the yield with *hsdMS*-2-modified pSK236 was significantly higher than that of pSK236 from *E*. *coli* K12 in MW2 and USA300 LAC (p<0.05) (Fig. 4A and 4B, respectively). In the case of N315, transformation with pSK236 from *E*. *coli* K12 and *E*. *coli* DC10B yielded a large number of colonies (800–900), presumably due to a mutation in the *sauUSI* gene that constitutes the type IV restriction system in *S*. *aureus* [14]. Despite this, transformation with *hsdMS*-2-modified pSK236 was able to produce an even higher number of N315 transformants than pSK236 from *E*. *coli* K12 or DC10B (p<0.05 with the Student t test) (Fig. 4C). The results obtained with *hsdMS-2*-modified pEPSA5 were also consistent, with significantly higher numbers of MW2 and USA300 LAC transformants using the *hsdMS*-2-modified pEPSA5 than those from K12 or DC10B (Fig. 4D and 4E, respectively). In particular, transformation with pEPSA5 from *E*. *coli* K12 and DC10B yielded 50–100 transformants in MW2, but the efficiency was higher with *hsdMS*-2-modified pEPSA5 (Fig. 4D) (*p*<0.05 between *hsdMS*-2-modified pEPSA5 and pEPSA5 from *E*. *coli*). With USA300 LAC, however, transformation with pEPSA5 from *E*. *coli* K12 and DC10B hardly yielded any colonies whereas a similar procedure with *hsdMS*-2-modified pEPSA5 led to over 400 transformants (Fig. 4E). Direct transformation of N315 with any *E*. *coli* sourced pEPSA5 was greatly reduced (Fig. 4F) compared to similar transformations using pSK236 (Fig. 4C). Contrary to the general trend, however, transformation of N315 with *hsdMS*-2-modified pEPSA5 did yield a higher average number of colonies than those from K12 or DC10B, but the difference did not reach statistical significance.

## Discussion

The type I and IV R-M systems of *S*. *aureus* create barriers that prohibit easy transformation by shuttle vectors from *E*. *coli*, limiting the genetic manipulation of many clinical isolates, especially those that harbor multiple antibiotic resistance markers. By modifying *E*. *coli* DC10B cells to bypass not just one, but both these systems, our study here demonstrates that direct transformation of *S*. *aureus* strains with *E*. *coli* plasmids can be accomplished with ease.

*E*. *coli* DNA that is properly methylated at the appropriate TRS is theoretically able to bypass the type I R-M barrier of *S*. *aureus*. We observed that the contribution of each *hsd* system to this barrier was not equal however, as the transformation of DNA from *E*. *coli* containing *hsdMS*-2 was the more efficient of the two systems. However, given that all known strains contain both *hsd* operons, it is conceivable that the combined activities of HsdMS-1 and HsdMS-2 together in a single *E*. *coli* strain could augment transformational efficiency further; this is an area that we are currently investigating.

We also observed that the type I *hsd* restriction and modification sites in CC5 may not be as well defined as previously determined [13]. Specifically, while there are no identified *hsdMS*-2 CC5 TRS motifs in pSK236 (Table 3), N315 (a CC5 strain) upon transformation with a plasmid from an *hsdMS*-2-containing *E*. *coli* strain yielded many more colonies than the analogous *hsdMS*-1-modified pSK236 (Fig. 2C). Therefore, it appears that the presence, absence or frequency of the TRS sequences cannot completely explain the difference in transformation efficiencies observed between the *hsdMS*-1 and *hsdMS*-2 systems.

Interestingly, we observed variances in the transformation efficiencies of the two plasmids among the tested *S*. *aureus* strains based on their clonal complex. Using the same amount of plasmid, *hsdMS*-2-modified pSK236 was more readily transformed into all three *S*. *aureus* strains compared to *hsdMS*-2-modified pEPSA5, with the difference most pronounced in N315 and USA300 LAC. Besides the possibility of variance in the TRS systems of N315 as noted above, one plausible explanation for the difference in transformation efficiency is that pSK236 and pEPSA5 may have differential cytosine methylation by the HsdMS-2 complex. Another possibility is that methylation of adenines by *dam* in *E*. *coli* DC10B may interfere with the methylation by the HsdMS systems. Alternatively, both HsdMS complexes and/or other as-yet-undescribed factor(s) may be required to augment transformation efficiency of pEPSA5 in our strains. The end result is likely improper methylation of the plasmid DNA, ultimately causing their restriction at sites recognized by the *S*. *aureus* type I R-M R_2_M_2_S complex [34].

There also appears to be a difference in the transformation efficiency among *S*. *aureus* strains using the same plasmid. N315 has been reported to have a faulty *sauUSI* gene [14], which limits the ability of the strain to recognize and restrict foreign DNA based on type IV R-M. Thus, unmodified *E*. *coli* K12 pSK236 DNA, typically susceptible to *S*. *aureus* R-M type I and IV barriers, was able to bypass the type IV R-M barrier to yield *S*. *aureus* N315 transformants at the same rate as that isolated from *E*. *coli* DC10B cells. However, when pSK236 DNA was modified to bypass both types I and IV restriction barriers, transformation of N315 was even more efficient than plasmid DNA from *E*. *coli* DC10B. Interestingly, this same strain that proved to be efficient for pSK236 appeared to be very difficult for transformation with pEPSA5. While we could still obtain a few transformants with *hsdMS*-2-modified pEPSA5, the transformation efficiency dropped further with unmodified or *E*. *coli* DC10B-derived plasmid (Fig. 4F). This finding suggests that there may be a fundamental difference in the way the two plasmids are recognized by the type IV R-M system or that another restriction barrier is at play. Given that the G-C content of pEPSA5 is similar to that of pSK236 (37.1% vs. 39.5%), the reason for this discrepancy remains unclear. Remarkably, we found an opposite scenario for the MW2 strain, wherein the transformation efficiency was moderate with pEPSA5 from *E*. *coli* K12 and DC10B, but the efficiency was even higher with *hsdMS*-2-modified pEPSA5.
